# O.4.4-5 Transition of the chronically ill patient to a physically active lifestyle: a DELPHI study with experts from the field of sport

**DOI:** 10.1093/eurpub/ckad133.202

**Published:** 2023-09-11

**Authors:** Edwin Mathéi, Alexandre Mouton, Axelle Petit, Gautier Horin

**Affiliations:** University of Liège, Belgium; edwin.mathei@hepl.be; University of Liège, Belgium; edwin.mathei@hepl.be; University of Liège, Belgium; edwin.mathei@hepl.be; University of Liège, Belgium; edwin.mathei@hepl.be

## Abstract

The purpose of this study is to gather the opinion of experts from the sports/association context on the theme of physical activity for chronically ill patients in the out-of-hospital context.

To conduct this study, we used the DELPHI methodology (Dalkey & Helmer, 1963). This method is used to reach a convergence of opinions on a subject with experts from the related field. This methodology consists in a succession of survey submitted to our population of sport experts (n = 62). Every survey is preceded by a feedback about the previous results: in that way experts can answer the following survey taking into account the opinion from the majority of respondents. Surveys have been sent to the expert electronically using SurveyMonkey®. This study contains three consecutive surveys (rounds). Each survey has been validated by the *Content Validity Index* (CVI) methodology to assess the relevance and the clarity of statements (Rubio et al., 2003).

Each steps of the DELPHI process have been segmented in six sections: participants, stakeholders, communication/transition, type of activity, financial aspects and motivation. After the three rounds of the DELPHI a consensus has been reached for every section of the survey (more than 60% of approval on the topic). 81% of the experts agreed that assessments were needed for participants before taking part in sport activities. 63% of the experts agreed that stakeholders needed at least a bachelor’s degree in sport sciences to supervise activities. 75% agreed that the groups must be rather small (4-5 persons). 78% agreed that participants have to pay, at least partly, for their participation. 81% agreed that social interactions are the main motivational factor of out-of-hospital sport activity among those participants.

Despite several limitations, this study led to encouraging results concerning the development of physical activities for chronically ill patient in out of hospital context. We believe that these results will enable sports associations to have a basis for developing “sport-health” activities, tailored to the local context. In the other hand, we also believe that our research will raise awareness of the role of local specialists in physical activity promotion among this type of population.

